# SARS-CoV-2 leads to myocardial injury in rhesus macaque

**DOI:** 10.1038/s41392-021-00747-5

**Published:** 2021-09-06

**Authors:** Yufan Feng, Xiaomin Song, Yongfa Huang, Wei Deng, Man Li, Xiaoxiao Guo, Chuan Qin, Wei-Min Tong, Jiangning Liu, Jing Wang

**Affiliations:** 1 Institute of Basic Medical Sciences, Chinese Academy of Medical Sciences, School of Basic Medicine Peking Union Medical College, Beijing, China; 2 Department of Cardiology, Peking Union Medical College Hospital, Peking Union Medical College and Chinese Academy of Medical Sciences, Beijing, China; 3 NHC Key Laboratory of Human Disease Comparative Medicine, Beijing Key Laboratory for Animal Models of Emerging and Remerging Infectious Diseases; Institute of Laboratory Animal Science, Chinese Academy of Medical Sciences and Comparative Medicine Center, Peking Union Medical College, Beijing, China; 4 Department of Pathology, Beijing Ditan Hospital, Capital Medical University, Beijing, China; 5 Department of Pathology, Institute of Basic Medical Sciences, Chinese Academy of Medical Sciences and Peking Union Medical College; Molecular Pathology Research Center, Chinese Academy of Medical Sciences, Beijing, China

**Keywords:** Cardiovascular diseases, Infectious diseases

**Dear Editor**,

Coronavirus disease 2019 (COVID-19) is caused by severe acute respiratory syndrome coronavirus 2 (SARS-CoV-2) infection, which induces multiple cardiovascular complications including acute myocardial injury resulting from acute coronary syndrome, myocarditis, stress-cardiomyopathy, arrhythmias, cardiogenic shock, and cardiac arrest. The incidence of myocardial injury in COVID-19 patients was up to 20% or higher in the subpopulation with existing cardiovascular diseases, accounting for 69.4% of mortality.^1^ Although the presence of angiotensin-converting enzyme 2 (ACE2) in cardiomyocytes could result in viral myocarditis in COVID-19 patients, the cardiopathology involved in COVID-19 is still unclear.

We aimed to explore the mechanism of myocardial injury in COVID-19 patients using a rhesus macaque model of SARS-CoV-2 respiratory tract infection. First, we analyzed previously published single-nucleus RNA sequencing data of the normal atrial tissue of 16 monkeys.^2^ As shown in Supplementary Fig. S1, the main cell types were defined based on published marker genes.^2^ Furthermore, the expression of *ACE2* and the serine protease *TMPRSS2* were investigated. *ACE2* was expressed in cell types including pericytes, epicardial cells, and cardiomyocytes. *TMPRSS2* was expressed in cells of the cardiac conduction system, endothelial cells, and cardiomyocytes (Fig. 1a). Next, we performed KEGG enrichment analysis using the differentially expressed genes between cells with and without *TMPRSS2* expression. Interestingly, among the enriched KEGG pathways, we found that genes up-regulated in the TMPRSS2-expressing cells (e.g., MX1, MX2, IFIH1, DDX58, EIF2AK2) were enriched in the Coronavirus-COVID-19 pathway (Fig. 1b), which includes the genes that are known to be involved in the invasion of SARS-CoV-2 into alveolar epithelial cells (https://www.kegg.jp/keggbin/show_pathway?hsa05171/TMPRSS2/MX1/PIK3R1/IFIH1/DDX58/EIF2AK2/ADAM17/MX2/JAK1). These results suggested that *TMPRSS2*-expressing cells, including cardiomyocytes, may be directly attacked by the virus, potentially causing viral myocarditis.

**Fig. 1:**
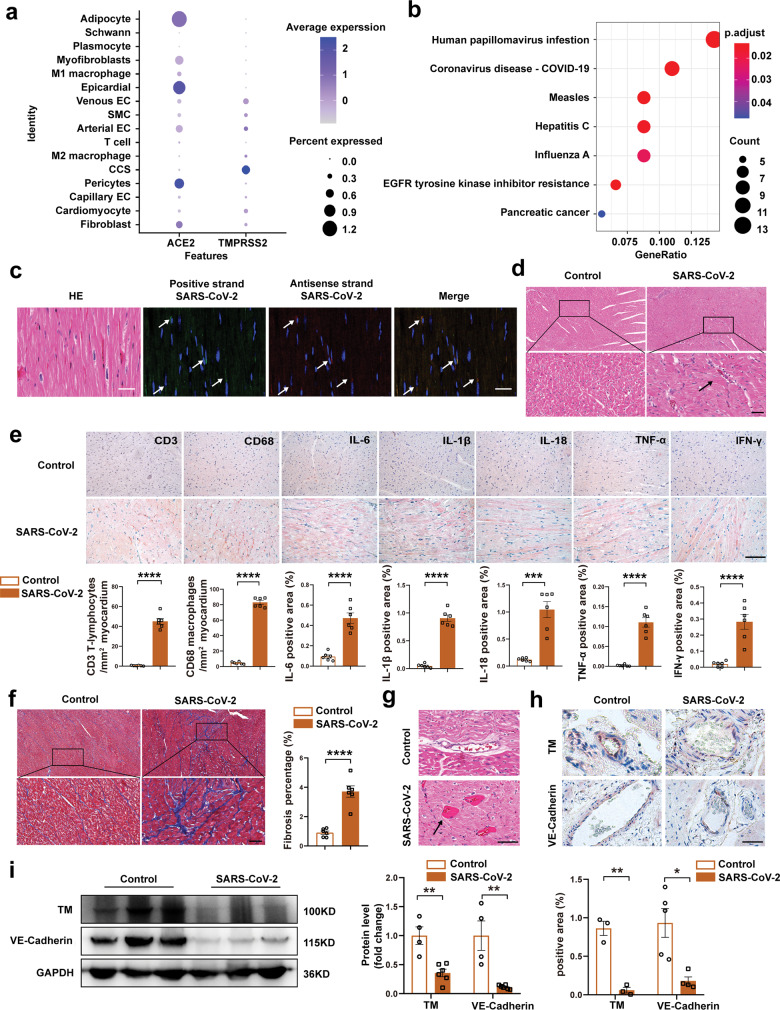
The mechanisms of cardiac injury caused by SARS-CoV-2. **a** Dotplot representing the relative expression of *ACE2* and *TMPRSS2* in the heart. Public single-nucleus RNA sequencing (snRNA-seq) data from the Genome Sequence Archive database (CRA002689) were used for bioinformatics analysis. **b** KEGG pathway enrichment analysis shows the pathways associated with *TMPRSS2*-expressing cells. **c** SARS-CoV-2 was visualized by RNA in-situ hybridization in the SARS-CoV-2 infection rhesus macaques. HE staining in the left. Positive strand SARS-CoV-2 was detected by probes labeled with green fluorescence. The antisense strand of SARS-CoV-2 was detected with probe labeled with red fluorescence, indicating the replication of virus (Scale bar = 50 μm). **d** Representative views of HE staining of the left ventricle. To simulate respiratory tract infection, rhesus macaques in SARS-CoV-2 group were intratracheally challenged with SARS-CoV-2 at 50% TCID50 of 1 × 10^6^. The arrow indicates the infiltration of inflammatory cells in SARS-CoV-2 group. (*N* = 6 in each group, Scale bar = 50 μm). **e** Representative immunohistochemical staining for CD3, CD68, IL-6, IL-1β, IL-18, TNF-α, and IFN-γ of the left ventricles and quantification of staining positive areas. (*N* = 6 in each group, Scale bar = 50 μm). **f** Representative images of Masson staining in the left ventricles of the two groups. (*N* = 6 in each group, Scale bar = 50 μm). **g** Representative views of HE staining of the left ventricle. The arrow indicates a micro-thrombus in SARS-CoV-2 group. **h** Representative immunohistochemical staining for TM and VE-cadherin of microvascular in the left ventricles. (TM: *n* = 3 in each group; VE-cadherin: *n* = 5 in control group, *n* = 4 in SARS-CoV-2 group, Scale bar = 50 μm). **i** Representative western blots showing TM and VE-cadherin protein expression in the two groups (*N* = 3 in each group). Protein levels were normalized to GAPDH protein. (*N* = 4 in control group, *n* = 6 in SARS-CoV-2 group). All data are presented as mean ± SEM. *p < 0.05, **p < 0.01, ***p < 0.001, ****p < 0.0001. The unpaired two-tailed student’s *t*-test was used to assess differences between the two groups. EC endothelial cells, SMC smooth muscle cells, CCS cardiac conduction system cell, HE hematoxylin and eosin, TCID50 tissue-culture infectious doses, TM thrombomodulin

To explore the possibility of viral myocarditis, six rhesus macaques (3–5 kg, 3–5 years of age) were intratracheally challenged on the first day with 1 × 10^6^ TCID50 (50% tissue-culture infectious dose) of SARS-CoV-2, and six age- and body weight-matched macaques served as healthy controls.^3^ Seven days post initial infection, the left ventricles were collected. As shown in Fig. 1c, SARS-CoV-2 positive- and negative-strand RNA was found in the infected macaques by RNA in-situ hybridization, indicating that SARS-CoV-2 infection and replication occurred in the ventricular tissue. Viral RNA copies were present at 1 × 10^1.2^ copies/g in the SARS-CoV-2 group.^3^ The presence of myocarditis was demonstrated by the higher numbers of invaded inflammatory cells in the virus-exposed group (Fig. 1d). Importantly, the typical histopathology of myocarditis with increased interstitial infiltration of CD68^+^ macrophages and CD3^+^ T lymphocytes was observed in the virus-exposed group, along with increased levels of inflammatory cytokines (Fig. 1e; Supplementary Fig. S2). In addition, cardiac inflammation was accompanied with sporadic cardiac fibrosis in SARS-CoV-2-infected rhesus macaques (Fig. 1f). These results demonstrated the occurrence of viral myocarditis after SARS-CoV-2 infection.

In addition to myocarditis, other etiologies may lead to cardiac injury. Endothelial dysfunction in response to viral infection can lead to coagulation disorder. The activation of the coagulation pathway with the possible development of disseminated intravascular coagulation (DIC) may result in thrombus formation in severe COVID-19 patients. Consistently, we found micro-thrombi in SARS-CoV-2-infected macaques (Fig. 1g) and decreased thrombomodulin (TM) and VE-cadherin (Fig. 1h, i), which promoted coagulation and increased endothelial permeability respectively. We also found increased expression of ICAM-1 and VCAM-1 in the SARS-CoV-2 group, suggesting the propensity for extravasation of inflammatory cells and subsequent myocarditis (Supplementary Fig. S3). Taken together, these results indicated that endothelial dysfunction was associated with thrombotic events and cardiac injury in COVD-19.

Besides cardiomyocytes and endothelial cells, our results showed that T cells, pericytes, and fibroblasts also express *ACE2* and *TMPRSS2* (Fig. 1a) and may participate in cardiac injury in COVID-19. Activated inflammatory response by T cells may contribute to myocardial dysfunction. *ACE2* is highly expressed on pericytes, allowing SARS-CoV-2 to attack these cells and contribute to cardiac damage. Fibroblasts secret collagens and extracellular matrix proteins to promote cardiac fibrosis. In addition, our results showed that some cells express *ACE2* but not *TMPRSS2*, including M1 macrophages and epicardial cells. M1 macrophages mediate inflammatory response via producing pro-inflammatory cytokines in heart failure. Epicardial cells participate in the development of coronary artery disease and atrial fibrillation via structural and electrical remodeling of the atria. This suggests multiple cell types with or without *TMPRSS2* may contribute to the cardiac damage in COVID-19.

However, the molecular mechanism of myocardial injury in COVID-19 remains to be elucidated. MAPK, NF-κB, and PI3K signaling pathways induce a myocardial inflammatory response and cardiomyocytes apoptosis in viral myocarditis. Inhibition of MAPK alleviates cardiac function in mice with viral myocarditis. The augmented inflammation of viral myocarditis is mainly associated with NF-κB signaling. PI3K signaling participates in cardiomyocyte autophagy in CVB3 (Coxsackievirus B3) infection.^4^ Taken together, these findings indicate that MAPK, NF-κB, and PI3K signaling are involved in myocardial injury and deserve further investigation in SARS-CoV-2-induced myocardial injury.

Previous studies have demonstrated that cardiomyocytes are directly infected by SARS-CoV-2 in vitro.^5^ Human induced pluripotent stem cell-derived cardiomyocytes (iPS-CMs) are susceptible to SARS-CoV-2 infection via ACE2, and pathologic responses including apoptosis, oxidative stress, and immune response were identified in iPS-CMs challenged with SARS-CoV-2.^5^ Furthermore, SARS-CoV-2 can infect cardiomyocytes in 3D cardiac tissue environment and living human cardiac tissue slices.^5^ In this study, using SARS-CoV-2-infected rhesus macaques, we demonstrated the occurrence of viral myocarditis in vivo for the first time.

This particular animal model and time point were chosen to mimic the clinical signs of COVID-19 patients. At seven days post infection, viral RNA copies (~1 × 10^4^ copies/mL) were detected in the lungs, which also showed ground-glass opacity and obscure markings in the upper lobe of the right lung by X-ray.^3^Our findings on SARS-CoV-2-induced cardiac injury are particularly important, given that this animal model is used for vaccine testing and drug development.

In conclusion, we found that SARS-CoV-2-infected rhesus macaques showed viral myocarditis, where inflammation and endothelial injury jointly resulted in cardiac damage. Our results suggest that the possibility of cardiac injury requires close monitoring in COVID-19 patients, particularly in those with pre-existing cardiovascular conditions. The potential myocardial damage caused by future therapeutic agents deserves careful investigation.

## Supplementary information

supplemental material

## Data Availability

All data in this article and supplementary materials are publicly available to authorized users.
