# Supplementary material for: SARS-CoV-2 leads to myocardial injury in rhesus macaque
Source: Signal Transduct Target Ther. 2021 Sep 6;6:338. doi: 10.1038/s41392-021-00747-5 (PMC8419658; doi:10.1038/s41392-021-00747-5)
Supplement: Supplementary file 1 — supplemental material [file 41392_2021_747_MOESM1_ESM.docx]

Supplementary Materials for

SARS-CoV-2 leads to myocardial injury in rhesus macaque

Yufan Feng, Ph.D.^1*^; Xiaomin Song, Ph.D.^1*^; Yongfa Huang, B.S.^3*^; Wei Deng, Ph.D.^2^; Man Li, M.D.^4^; Xiaoxiao Guo, M.D.^3^; Chuan Qin, M.D.^2^; Wei-Min Tong, M.D.^5^; Jing Wang, Ph.D.^1#^; Jiangning Liu, Ph.D.^2#^.

# Co-correspondence to: [liujn@cnilas.org](mailto:liujn@cnilas.org), wangjing@ibms.pumc.edu.cn

**This file includes:**

Figures. S1 to S3

Captions for Figures. S1 to S3


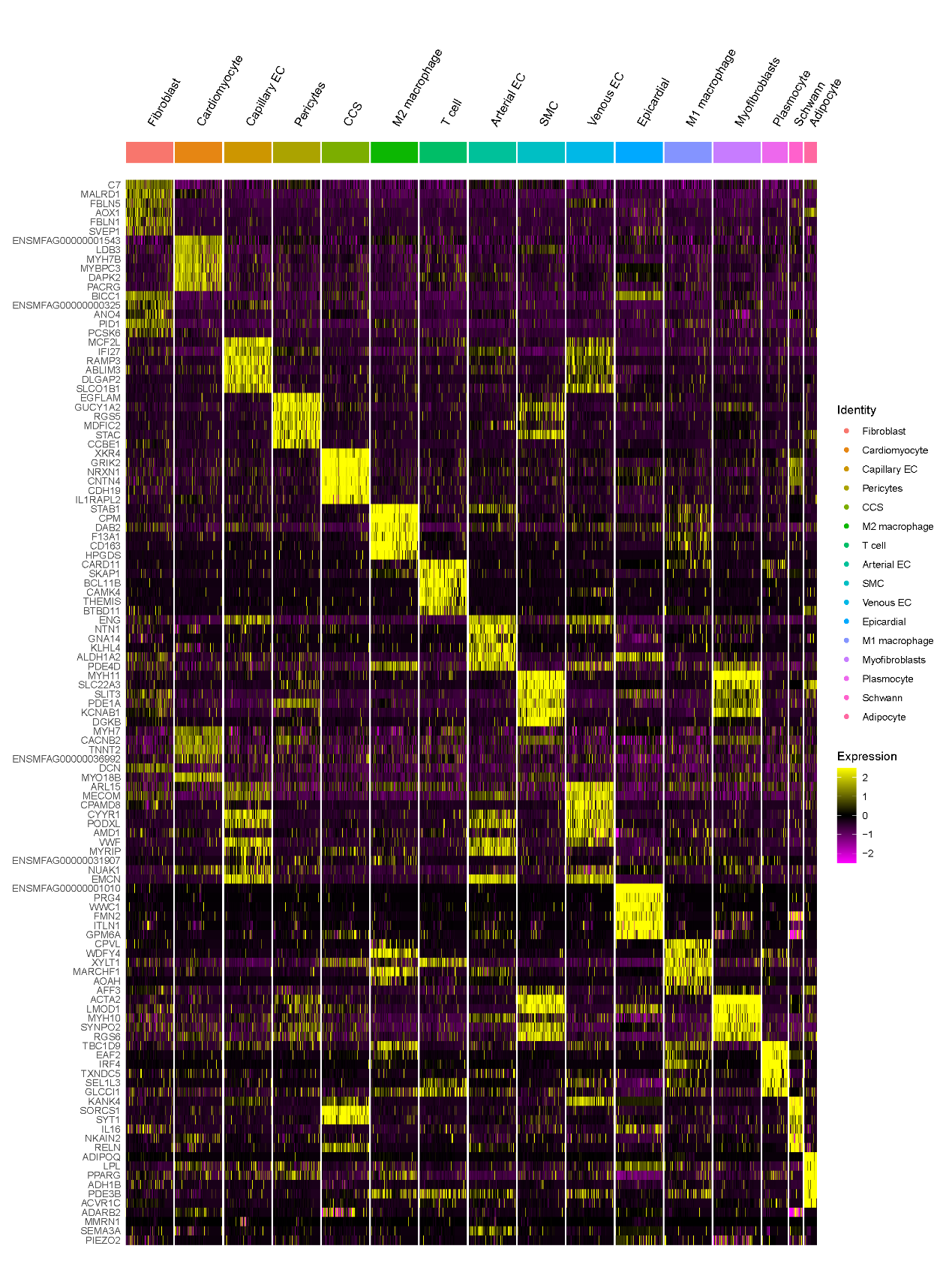


**Figure. S1. Heatmap of the marker genes in each cluster.** Heatmap showing the expression levels of the marker genes in each cluster from the hearts. Each column represents a cell cluster, which is defined as a cell type based on the top six highly expressed genes (ranked by logFC) compared to the other cell clusters. The rows exhibit the relative expression (normalized and scaled by z-score) of the marker genes.


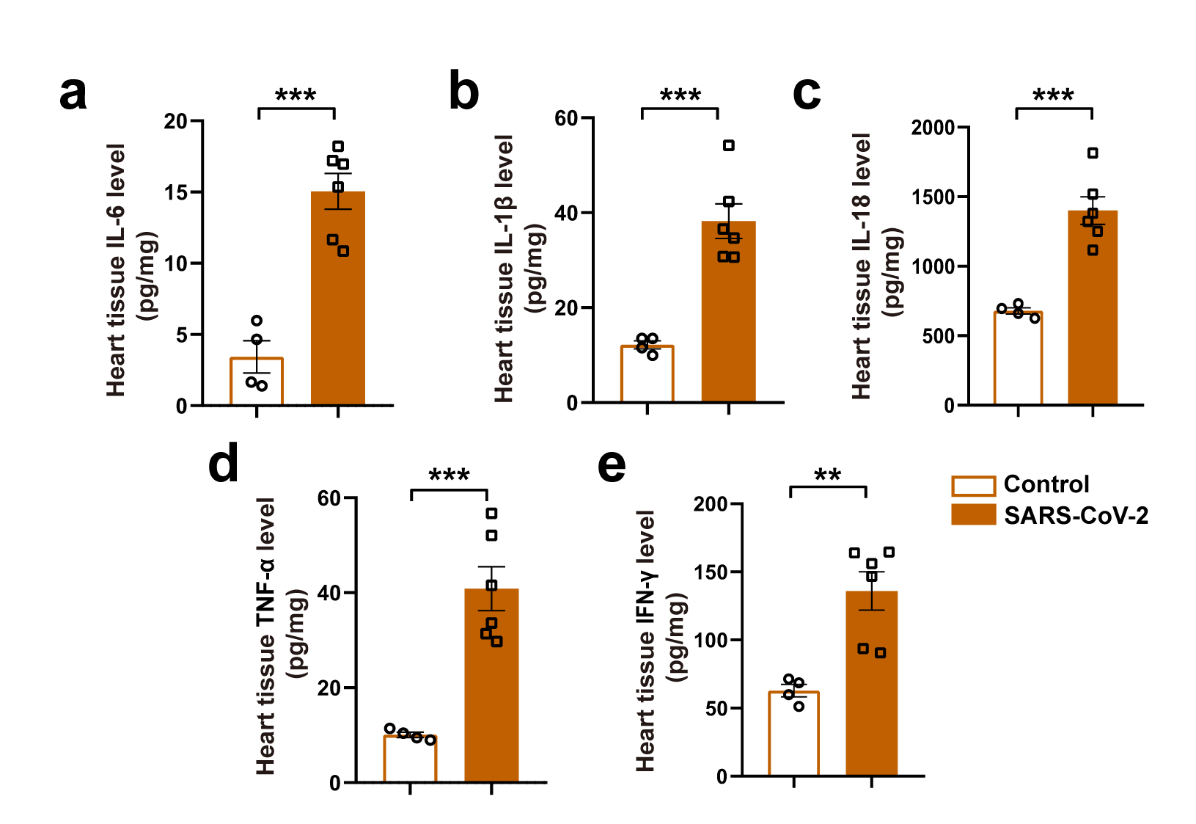


**Figure. S2. Expression of inflammatory cytokines in the left ventricles.** ELISA was carried out to analyze the expression of the inflammatory cytokines. **a.** The level of IL-6. **b.** The level of IL-1β. **c.** The level of IL-18. **d.** The level of TNF-α. **e.** The level of IFN-γ.


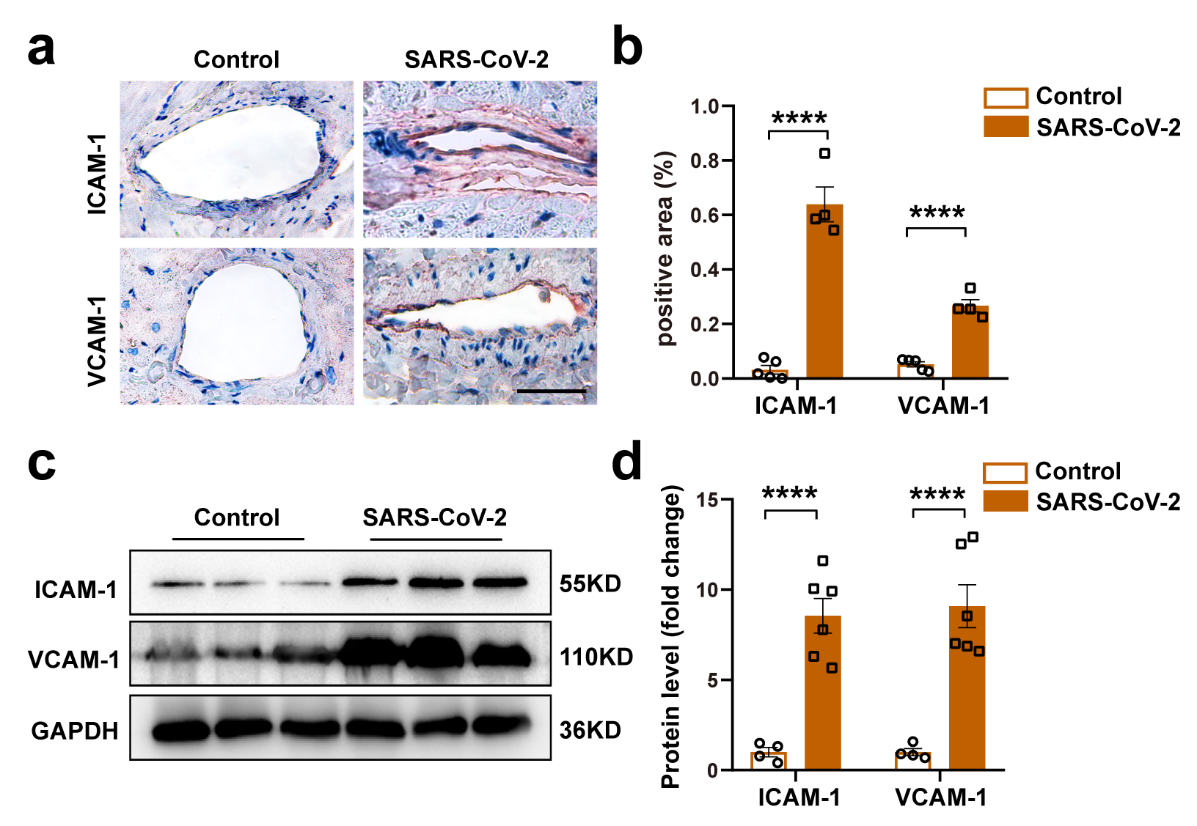


**Figure. S3. Expression of ICAM-1 and VCAM-1. a.** Representative immunohistochemical staining for ICAM-1 and VCAM-1 of microvascular in the left ventricles. (ICAM-1, VCAM-1: n = 5 in control group, n = 4 in SARS-CoV-2 group, Scale bar = 50 μm). **b.** The positive area in each group was analyzed. **c.** Representative western blots showing ICAM-1 and VCAM-1 protein expression in two groups (N = 3 in each group). **d.** Protein levels were normalized to GAPDH protein. (N= 4 in control group, n = 6 in SARS-CoV-2 group).
